# Coexistence between wildlife and livestock is contingent on cattle density and season but not differences in body size

**DOI:** 10.1371/journal.pone.0236895

**Published:** 2020-07-31

**Authors:** Keenan Stears, Adrian M. Shrader

**Affiliations:** 1 Department of Ecology, Evolution and Marine Biology, University of California Santa Barbara, Santa Barbara, CA, United States of America; 2 South African Environmental Observation Network, Ndlovu Node, Scientific Services, Kruger National Park, Phalaborwa, South Africa; 3 University of KwaZulu-Natal, Scottsville, South Africa; 4 Mammal Research Institute, Department of Zoology and Entomology, University of Pretoria, Pretoria, South Africa; Sichuan University, CHINA

## Abstract

Many studies on the coexistence of wildlife with livestock have focused primarily on similar-sized species. Furthermore, many of these studies have used dietary overlap as a measure of potential competition between interacting species and thus lack the important link between dietary overlap and any negative effects on a particular species–a prerequisite for competition. Consequently, the mechanisms that drive interspecific interactions between wildlife and cattle are frequently overlooked. To address this, we used an experimental setup where we leveraged different cattle stocking rates across two seasons to identify the drivers of interspecific interactions (i.e. competition and facilitation) between smaller-bodied oribi antelope and cattle. Using direct foraging observations, we assessed dietary overlap and grass regrowth, and also calculated oribi nutritional intake rates. Ultimately, we found that cattle compete with, and facilitate, smaller-bodied oribi antelope through bottom-up control. Specifically, cattle facilitated oribi during the wet season, irrespective of cattle stocking density, because cattle foraging produced high-quality grass regrowth. In contrast, during the dry season, cattle and oribi did not co-exist in the same areas (i.e. no direct dietary overlap). Despite this, we found that cattle foraging at high densities during the previous wet season reduced the dry season availability of oribi’s preferred grass species. To compensate, oribi expanded their dry season diet breadth and included less palatable grass species, ultimately reducing their nutritional intake rates. Thus, cattle competed with oribi through a delayed, across-season habitat modification. We show that differences in body size alone may not be able to offset competitive interactions between cattle and wildlife. Finally, understanding the mechanisms that drive facilitation and competition are key to promoting co-existence between cattle and wildlife.

## Introduction

The debate over whether livestock, primarily cattle (*Bos* spp.), and native wildlife compete with each other for resources has been ongoing for decades. However, definitive evidence of these competitive interactions, and whether these interactions are responsible for the observed decline in wildlife numbers, is rather limited [1, 2]. This is primarily due to the difficulty in determining whether a shared resource is indeed limited [1]. The vast majority of the literature only provides evidence for potential competition via dietary overlap [3–5], with only a handful providing evidence of actual competition with one species having a deleterious effect on another–an integral component of competition (e.g. [6–9]). Thus, experimental studies that provide direct evidence for competition, and the factors driving these interactions, between livestock and wildlife are crucial for the conservation of wildlife populations outside of protected areas, or in areas where cattle feed within protected areas [1, 10, 11].

Body size is an important trait involved in resource partitioning and interspecific interactions because a herbivore’s morphology, physiology, and ecology (e.g. gut capacity, bite size, food intake rate, and feeding site selection) vary allometrically with body size [12–15]. Thus, body size ratios are important predictors for potential resource competition, which would explain why the majority of livestock-wildlife studies have focused on the interactions of similar-sized herbivores where the potential for resource overlap is greatest [8, 16–18].

Owen-Smith [19] modeled the combined effect of differences in body size and relative bite dimension and found that different grazers specialized on different grass height categories, which allowed for coexistence. Similarly, Prins and Olff [14] predicted that with coexisting grazer assemblages, there should be an optimum difference in body mass, with each grazer species being, on average, a constant proportion larger than the closest smaller grazer. However, Arsenault and Owen-Smith [20] suggest that the scaling of mouth-width relative to body size may be more important in explaining grass height selection than body size. Despite the differential use of resources imposed by body size and mouth morphology, the findings of empirical studies on foraging between herbivores of different body sizes, and the nature of the interspecific interactions between these herbivores, does not always match theoretical expectations. For example, larger grazers can compete with smaller grazers by reducing food availability, especially during the dry season (i.e. exploitation competition; [21–23]), or through more long-term negative effects (i.e. habitat modification; [1]). Large grazers can also influence grassland productivity via their foraging by promoting high-quality grass regrowth, which may positively influence (facilitate) smaller grazers [24–27]. However, through their selective foraging, small herbivores may outcompete larger herbivores by reducing the availability of high-quality green grass during the dry season [28].

As wildlife continues to experience increased pressure from livestock [29], the above examples highlight the necessity to understand how cattle interact with a diverse native herbivore assemblage and not merely herbivores of a similar size. Furthermore, understanding the nature and processes that mediate interspecific interactions between different-sized herbivores can ensure the successful management and coexistence of mixed wildlife and livestock grazing assemblages. Ultimately, whether interspecific interactions promote coexistence, or increase competition, depends on herbivore density [18], season [8], and the biology of the interacting species [30, 31].

**Fig 1:**
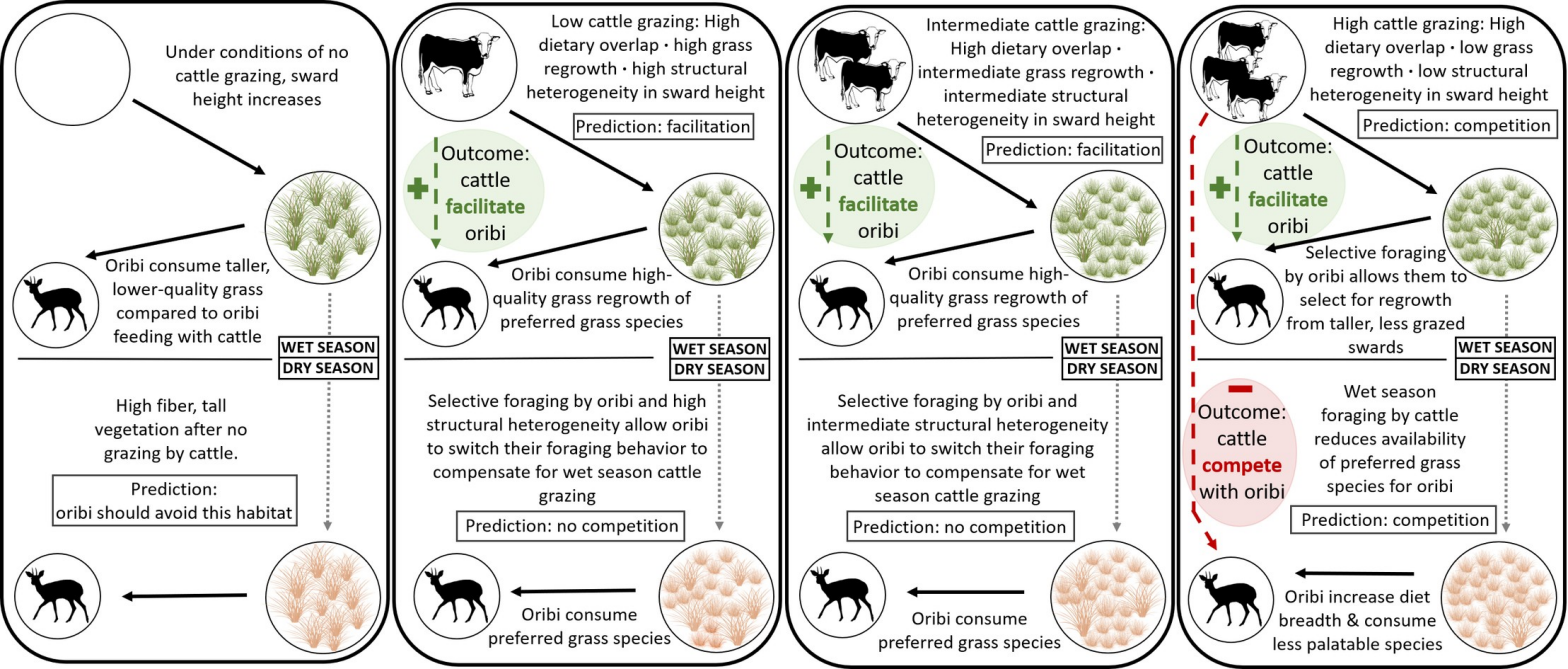
Graphical representation of our experimental design. We leveraged different cattle stocking rates across two seasons (wet and dry) to identify potential drivers of interspecific interactions between oribi antelope and cattle. Cattle grazing under different intensities can alter the structural heterogeneity of grass swards as well as alter the availability of specific grass species. In turn, these changes can influence oribi foraging behavior and ultimately, influence their nutritional intake rates, which can lead to either competitive or facilitative interactions between cattle and oribi. Cattle and oribi only directly overlap during the wet season. As a result, interspecific interactions that are observed during the dry season as a consequence of the intensity of cattle grazing in the previous wet season.

In South Africa, oribi antelope (*Ourebia ourebi*) provide a unique opportunity to explore interspecific interactions between domestic and wild herbivores because the majority of oribi populations occur on private rangelands resulting in frequent interactions between oribi and cattle [32]. Furthermore, both species are primarily grazers and the ~40-fold difference in body size between these two species allowed us to examine the role of body size in interspecific interactions. In South Africa, oribi are listed as endangered and population declines have been attributed to competition with cattle [32, 33]. However, there is no empirical evidence to support this. We address this issue through an experimental study exploring whether interspecific resource competition occurred between livestock and oribi. To do this, we explored how cattle foraging under different stocking rates influenced the seasonal foraging behavior, crude protein intake, and digestibility of vegetation consumed by oribi (see Fig 1). Further objectives of the study were to identify potential mechanisms driving interspecific interactions and discuss the roles of body size and feeding ecology in determining the differential effects of livestock stocking rates on oribi populations. Finally, we discuss the implications of our results for the conservation of small-bodied wild herbivores that frequently interact with cattle on African rangelands and communal grazing areas.

## Methods

### Study area

We conducted our study on Arundel Farm (~770 ha) in Ixopo, South Africa (S 30°11.557 E 30°12.199) during the dry (June–August 2013) and wet (January–February 2014) seasons. Mean rainfall for the area was 725 mm (1995–2013; South African Weather Services). Arundel farm is stocked with ~370 head of beef cattle (Bonsmara breed) that are managed using a continuous grazing policy and are not herded. Arundel Farm is split into wet and dry season foraging areas. The wet season foraging area is divided into three fenced grazing areas (hereafter referred to as “camps”) totaling ~380 ha of natural grassland. These three camps also contained ~21 oribi. Within each camp, oribi were free-ranging and foraged within and around the cattle herds. During the dry season (May–September), the cattle are moved into the adjacent dry season foraging area (~380 ha). As a result, cattle and oribi only occur in the same camps, and thus directly interact, during the wet season. In addition to the wet and dry season foraging areas, there was a single fenced camp (~15 ha) that was ungrazed by cattle during our study period. This camp was adjacent to the wet season foraging area and was only accessible to and used by oribi. We only observed oribi feeding in this area during the wet season.

We calculated the cattle stocking rate for each camp as: land area/number of animal units (i.e. ha per AU). One adult beef cow (~650 kg) equaled one animal unit, and a heifer as 0.7 animal units [34]. Cattle stocking rates for the different camps were: 1.7 ha per AU, 1.5 ha per AU and 0.95 ha per AU, (hereafter referred to as “low”, “intermediate”, and “high” respectively). These stocking rates fall within the approximate stocking rates that are recommended for continuous grazing in the region ([i.e. summer grazing only: 1–2 ha per AU; [35]). In addition to cattle, each of the three camps had several resident oribi (low stocking rate camp: n = 7 individuals across three herds, intermediate stocking rate camp: n = 8 individuals across two herds, and high stocking rate camp: n = 6 individuals across two herds). The University of KwaZulu-Natal and Ezemvelo KZN Wildlife approved all aspects of the research design (Ethics code: 058/14/Animal and W/2052/01).

### Data collection

To determine seasonal changes in grass sward structure and grass greenness, we walked two, 1 km transects (separated by approximately 50 m to ensure data were spatially independent) through each camp in each of the five months. Within each camp, the starting location for each monthly transect was randomly selected to ensure the same areas were not resampled and thus, provide a random representation of sward height in each camp. Every 50 m along these transects, we randomly placed two quadrats (0.4 m^2^). Within each quadrat (n = 1230), we measured the sward height of the dominant grass species using the direct measurement method (a single measurement that represents the average sward height; [36]) and the percentage of green grass using Walker’s [37] eight-point scale (0%, 1–10%, 11–25%, 26–50%, 51–75%, 76–90%, 91–99%, and 100%). Prior to analysis, we combined these estimates into four greenness categories: very brown (0–10%), mainly brown (11–50%), mainly green (51–90%), and very green (91–100%).

Structural heterogeneity facilitates the coexistence of herbivores and is particularly important for oribi habitat selection [38]. As a result, we calculated the coefficient of variation (CV = (standard deviation/mean) x 100%) of grass sward height as a measure of structural heterogeneity per camp. The greater the variation in sward height, the greater the CV. We used the transect data for each month to calculate average seasonal heterogeneity values for each camp.

#### Grass regrowth

To determine if cattle grazing intensity influenced grass regrowth, and therefore the ability of cattle to compete with or facilitate oribi, we conducted a grass regrowth trial from 19 January to 25 February 2014. In each grazing area with different cattle stocking rates (i.e. camps), we selected 30 swards of *Hyparrhenia hirta*, which was the most consumed grass species by oribi (Table 1). All swards were similar in size (~8 cm in diameter) and were selected so that they formed a 6 x 5 grid, with each sward separated by ~1.5 m. To ensure we measured the same swards, we marked them with an orange nail hammered flush to the ground. To obtain an estimate of sward height, we averaged five height measurements of each sward every three days. For each camp, we determined: 1) mean net relative regrowth (i.e. (ln final sward height–ln initial sward height); [39]), 2) mean sward height at the end of the experiment, and 3) mean number of times a sward was grazed (see below).

**Table 1:** Dietary contribution of consumed grass species for cattle (wet season only) and oribi (*Ourebia ourebi*) antelope (wet and dry season) at low (1.7 ha/AU), intermediate (1.5 ha/AU), and high cattle stocking rates (0.95 ha/AU). Spatial and temporal overlap between the two herbivores only occurred during the wet season so the dietary overlap reflects the overlap between the top contributing grass species in the wet season diet of cattle and oribi.

	Low stocking rate			Intermediate stocking rate			High stocking rate		
	Dietary contribution (%)			Dietary contribution (%)			Dietary contribution (%)		
Grass species	Wet	Wet	Dry	Wet	Wet	Dry	Wet	Wet	Dry
	cattle	oribi	oribi	cattle	oribi	oribi	cattle	oribi	oribi
*Hyparrhenia hirta*	56.07	63.92	88.69	36.86	79.95	72.43	11.21	35.29	57.68
*Paspalum dilatatum*	9.82	10.82	3.62	0	0.55	8.5	0	0	0
*Setaria nigrirostris*	0	10.57	0	0	4.95	0	6.36	36.65	0
*Themeda triandra*	5.2	6.44	2.71	10.2	12.09	6.45	25.15	21.72	24.72
*Paspalum scrobiculatum*	10.4	2.06	0	6.27	0	0	0	0	0
*Tristachya leucothrix*	0	1.8	0	7.06	0	0	0	0.9	0
*Chloris gayana*	0	1.55	0.9	0	0	1.47	0	0	0
*Cyprus sedge*	0	0.77	0	0	0	0	0	0	0
*Eragrostis plana*	0	0.77	0	0	0	0	0	0	0
*Unknown succulent forb*	0	0.77	0	0	0	0	0	0	0
*Pennisetum clandestinum*	0	0.52	4.07	14.9	0	6.74	0	0	0
*Aristida junciformis*	8.09	0	0	5.49	0	0	0	0	0
*Unknown flat leaf forb*	0	0	0	0	2.47	0	0	0	0
*Digitaria eriantha*	0	0	0	0	0	0	0	2.71	0
*Setaria sphacelata var torta*	0	0	0	0	0	0	42.12	1.36	0
*Sporobolus africanus*	5.78	0	0	0	0	0	7.88	1.36	2.25
*Eragrostis curvula*	0	0	0	0	0	1.17	0	0	0.74
*Heteropogon contortus*	0	0	0	0	0	3.23	0	0	14.23
*Cymbopogon excavatus*	0	0	0	0	0	0	0	0	0.37
Dietary overlap	90%			77%			75%		

Because each stocking rate camp was unreplicated, we were unable to discern whether the observed differences in the above grass regrowth was a result of cattle grazing intensity or site-specific differences between camps. To account for this, we conducted a concurrent clipping experiment in the adjacent camp that was ungrazed by cattle. Because our goal was to make comparisons between the clipping experiment and the grazing grass regrowth trial at different stocking rates, we used the same grass species (*H*. *hirta*) and sward diameter selected in the grazing grass regrowth trial. For the clipping experiment, we used a blocked experimental design (n = 3 blocks), with each block (4 x 10 grid of grass swards) containing all four defoliation intensity treatments (i.e. each treatment had 10 replicates per block). The clipping treatments included: no clipping, one clip every two weeks (i.e. 3 clipping events during the experiment), one clip every week (5 clipping events), and two clips a week (10 clipping events). We clipped each sward to a height of 8 cm above the ground, which was the average height to which cattle reduced grass swards post grazing (pers. obs.). Each sward was marked (as above) and measured every three days (i.e. grass swards in the regrowth and clipping experiment were measured on the same days). For each clipping intensity, we determined the number of times a sward was clipped over the experimental period (set by our clipping frequencies), the relative regrowth, and the resulting sward height at the end of the experiment.

To compare the observed relative growth under different stocking rates to the expected growth rates due to variation in grazing pressure, we plotted net relative growth from the clipping experiment against the number of times the swards were clipped during the experiment (i.e. 3, 5, and 10). We used the linear relationship from the clipping experiment (see Data Analysis section below), and the relative regrowth to estimate the number of times a sward was grazed by cattle in a camp as a function of grazing pressure. The analysis showed a good qualitative agreement between the predicted (clipping experiment) and observed (grass regrowth in the cattle camps) relationships, thus, we are confident that the observed regrowth in each camp is a function of cattle grazing intensity.

#### Oribi and cattle foraging

To determine if and how cattle foraging affected oribi, we collected foraging data from both oribi and cattle in the early mornings and afternoons when oribi were most active [33]. We observed oribi from a stationary vehicle using binoculars and collected data from both sexes (8 males, 13 females). To reduce potential observation error or bias associated with conducting fine-scaled behavioral observations, all data were collected by the same trained observer throughout the study.

We initiated data collection when an individual oribi started feeding. Each foraging observation spanned five feeding steps (i.e. feeding stations; [40]). Preliminary observations indicated that the average area in which oribi fed before taking a step was ~0.4 m^2^. Thus, we represented each feeding station using a 0.4 x 0.4 m quadrat. Once the oribi had moved off by ~50 m, we approached the feeding stations on foot. For each foraging observation, we calculated the bite rate by dividing the total number of bites along the five feeding steps by the time taken for these bites. Additionally, we determined the bite mass of each bite by hand plucking a simulated bite from surrounding un-grazed grass of the same species, see [41]. Each of these bite mass estimates (n = 1802) were then dried at 60° C for 48 hours and weighed. For every foraging observation, we calculated dry matter intake rate (g/min) by multiplying the mean bite mass from the five feeding steps with the corresponding bite rate. In addition, for each bite, we recorded the plant species consumed, grass greenness, and used the surrounding ungrazed grass of the same species to estimate sward height of consumed vegetation, as per [42]. To determine mean sward height grazed by oribi for a feeding observation, we averaged the sward height of consumed vegetation for each bite in the five feeding stations. For each bite, we differentiated older use from newly foraged grass by the white appearance of the damaged cuticle, as per [42]. In total, we observed 39 oribi foraging observations in the wet season (low stocking rate camp: n = 14, intermediate stocking rate camp: n = 17, high stocking rate camp: n = 8) and 63 observations in the dry season (low stocking rate camp: n = 26, intermediate stocking rate camp: n = 15, high stocking rate camp: n = 23). For cattle, we followed a similar experimental protocol. However, for cattle, each feeding station was represented by a 1.5 x 1.5 m quadrat and we identified the grass species consumed, its height, and its grass greenness category. Comparisons of dietary overlap, sward height, and greenness of consumed vegetation by cattle and oribi occurred at the bite scale (oribi: low stocking rate camp: n = 364 bites, intermediate stocking rate camp: n = 388 bites, high stocking rate camp: n = 221; cattle: low stocking rate camp: n = 510 bites, intermediate stocking rate camp: n = 361 bites, high stocking rate camp: n = 990 bites) during the wet season (i.e. the period when both cattle and oribi foraged together in the camps).

#### Dietary overlap

The dietary contribution of the top contributing species (i.e. species that contributed >90% of the diet) to both oribi and cattle diets was determined within each feeding station (i.e. quadrat) for each foraging observation. We determined the wet season dietary contribution for each species by dividing the number of bites of that species by the total number of bites of all species in that time period. We calculated the dietary overlap at each stocking rate using Schoener’s index: *Ojk* = 1−1/2∑|*Pij*–*Pik*| where *O*_*jk*_ is the dietary overlap between ungulate species *j* and *k*; *P*_*ij*_ and *P*_*ik*_ are the utilization of the *i*th resource by the *j*th and *k*th species [43].

#### Nutritional intake rates of oribi

We analyzed the crude protein content (CP) and the organic matter digestibility (OMD) of the top six species comprising >90% of the diets in each stocking rate (see Results; Table 1) using Near Infrared Reflectance Spectroscopy (NIRS). Prior to analysis, all grass samples were oven dried at 60° C for 48 hours and milled. NIRS spectra were calibrated off a database of South African grasses that were analyzed using wet chemistry by the Wallon Agricultural Research Centre, Belgium. We were able to estimate measures of nutritional quality for each sample because the averaged standardized H value (distance between a sample and the centroid of the group) was lower than or close to 3.0 for each predicted parameter [44]. We estimated the nutrient concentration of bites by using the CP estimate of each grass species in the respective grass greenness category. For bites that did not contain one of the six chemically analyzed species, the mean nutrient concentration of the analyzed grasses for that grass greenness was assigned. The intake rate (g/min) of CP was determined by multiplying the nutrient concentrations of the grass by bite mass. We then established the mean nutrient concentration per foraging observation which was then multiplied by the respective bite rate to give the nutritional intake rate (g CP/min). We repeated the same procedure for OMD.

### Data analysis

To compare the average grass sward height in each stocking rate camp during the wet season, we used a Generalized Linear Model (GLM; Gamma distribution and Log link function). We ran another GLM (Gamma distribution and Log link function) to determine if wet season grazing by cattle influenced the average sward height during the dry season when cattle were not present. The dependent variable was sward height and the independent variable was stocking rate.

#### Grass regrowth

We modeled the relationship between the net relative regrowth and the number of times a sward was clipped during the clipping experiment (i.e. 3, 5, and 10 clips). We did not include the no clipping treatment because we were only interested in regrowth of swards that were grazed. The data were well fitted by a linear relationship, which yielded the following equation: y = −0.0606x + 0.6502, *r*^2^ = 0.89, where *y* is the relative regrowth and *x* is the number of times the sward was clipped during the experiment. The equation provides an estimate of the predicted net relative regrowth as a function of grazing pressure (i.e. how frequently a sward was eaten). To obtain an empirical estimate of how many times a sward was eaten per stocking rate camp, we used the above trend line to fit a relationship between stocking rate and relative regrowth (i.e. we used the relative regrowth in each stocking rate camp and solved for *x*). For statistical representation, the relative regrowth was back transformed into percentage growth.

We used a GLM (Gamma distribution and Log link function) to assess whether cattle grazing (independent variable) and the number of times a sward was grazed influenced the relative grass regrowth (dependent variable). Because these swards were grazed by cattle, the average net relative regrowth was negative for some swards. As a result, we transformed the data by adding the lowest negative relative regrowth value to each net relative regrowth value to remove all negative values from the dataset.

#### Dietary overlap between cattle and oribi

To determine if stocking rates (independent variable) influenced: 1) the sward height that cattle selected during the wet season, and 2) the sward height that oribi selected during the wet season (both dependent variables), we used GLMs with a Gamma distribution and Log link function. In addition, we ran a GLM (Gamma distribution and log link function) to determine if the wet season grazing by cattle (independent variable) influenced the sward height selection by oribi during the dry season (dependent variable) (i.e. inter-seasonal exploitation competition).

**Fig 2:**
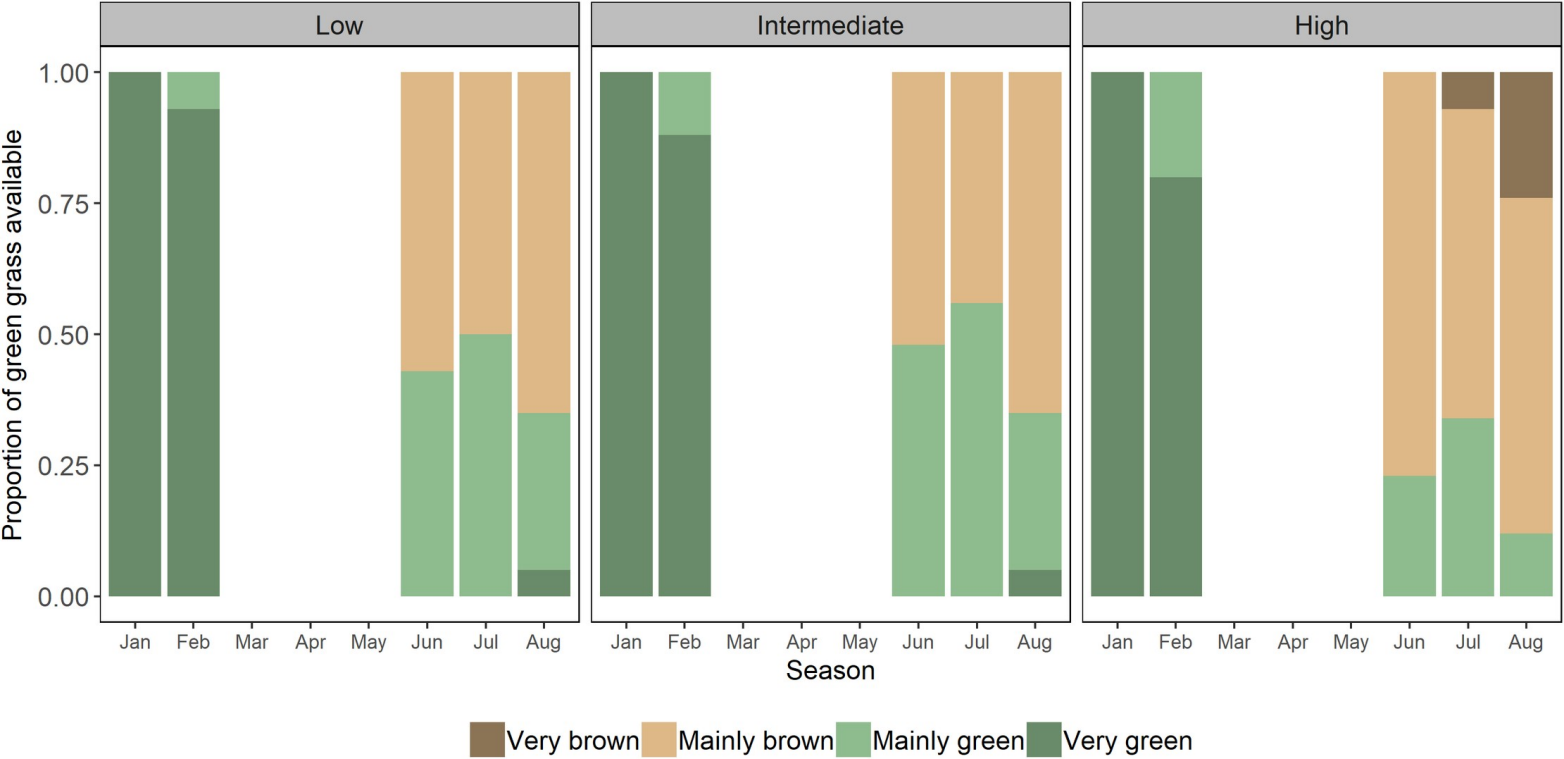
Availability of green grass. Seasonal differences in the availability of green grass in the different grass greenness categories for low, intermediate, and high cattle stocking rates (low: 1.7 ha per animal unit (AU); intermediate: 1.5 ha per AU; high: 0.95 ha per AU). Greenness categories are very brown 0–10%, mainly brown 11–50%, mainly green 51–90%, and very green 91–100%.

Stocking rate influenced the availability of green grass in the dry season (See Results and Fig 2). To determine if a reduced availability of green grass during the dry season influenced the proportion of green grass consumed by oribi, we calculated the proportion of green grass in their diet (dependent variable) among the different stocking rates. For each stocking rate, the number of bites in each grass greenness category was divided by the total number of bites obtained for that stocking rate camp. For this analysis, we used only the mainly green and very green categories because the oribi selectively fed from these greenness categories.

#### Nutritional intake rates of oribi

We ran a GLM (Gamma distribution and Log link function) to determine if different cattle stocking rates influenced; 1) the dry matter intake rate, 2) the CP intake rate, and 3) the OMD of consumed vegetation by oribi. We ran a separate model for each season (wet and dry) with dry matter intake rate, CP intake rate, and OMD as the dependent variable in their respective models, and stocking rate as the independent variable. Finally, we ran a GLM (Gamma distribution and Log link function) for CP and OMD (dependent variables) and compared these values between camps that were grazed and ungrazed by cattle (independent variable). We found no significant difference in CP intake rate and OMD across stocking rates (see below). As a result, we pooled the CP intake rates and OMD across the stocking rate camps for comparison with camps that were ungrazed by cattle. For the above analyses, we were unable to use mixed models (individual foraging observation as a random effect) because it was not possible to identify individual oribi and the foraging observations associated with a specific individual. All analyses were conducted in the R environment for statistical computing [45] and model assumptions were assessed using the *performance* package [46].

## Results

### Seasonal changes in sward height and the availability of green grass

During the wet season, the average sward height and resulting structural heterogeneity (measured as the coefficient of variation, CV, of grass sward height) was significantly influenced by cattle stocking rates (GLM: *χ*^2^ = 23.625, *df* = 2, *P* < 0.001), with similar measures in the low (23 cm, range 12–37 cm, 58% CV) and intermediate (24 cm, range 13–47 cm, 55% CV) camps, but much lower measures in the high stocking rate camp (18 cm, range 10–29 cm, 47% CV). Moreover, we found a carry-over effect where the increasing wet season stocking rates significantly reduced the average sward height and resulting structural heterogeneity in the dry season (GLM: *χ*^2^ = 121.107, *df* = 2, *P* < 0.001; low: 34 cm, range of 17–52 cm, 53% CV; intermediate: 34 cm, range 11–48 cm, 53% CV; high: 21 cm, range 12–30 cm, 46% CV).

During the wet season, the availability of green grass was similar among all camps, with grass being primarily very green and mainly green (Fig 2). However, as the seasons progressed, the availability of green grass decreased (Fig 2). In the dry season, the low and intermediate grazing camps had higher availabilities of green grass (35% and 32% respectively) than the high stocking rate camp (23%). In addition, the high stocking rate camp lacked very green grass, and was the only area containing very brown grass during the dry season.

### Grass regrowth

We found that over the duration of the grass regrowth trial (38-day observation period), swards in the low stocking rate camp were grazed ~4 times, those in the intermediate stocking rate camp were grazed ~3 times, and those in the high stocking rate camp were grazed ~14 times. As a result, the different grazing pressures influenced net grass regrowth (GLM: *χ*^2^ = 85.508, *df* = 2, *P* < 0.001). The low and intermediate camps had relatively similar positive regrowth (+46% and +56% respectively), whereas the high stocking rate camp showed negative overall growth (-20%).

### Dietary overlap between cattle and oribi

Throughout the study, both oribi and cattle maintained narrow diet breaths, with six species contributing to >90% of oribi diets and six species contributing to >85% of cattle diets (Table 1). These narrow diets resulted in a large dietary overlap between oribi and cattle across all stocking rates (low stocking rate = 90%, intermediate = 77%, high = 75%; Table 1). There was also a high degree of overlap between the wet season cattle diet and the dry season oribi diet (low stocking rate: 77% overlap, intermediate: 74% overlap, high: 70% overlap).

Comparing the dietary overlap of the top species consumed by oribi in the wet and dry seasons, we found that the greatest seasonal change in oribi diet (i.e. lowest overlap) was in the high stocking rate camp (62% overlap), compared to the low (75% overlap) and intermediate (83% overlap) stocking rate camps. Moreover, the high intensity grazing pressure from cattle during the wet season reduced the availability of preferred grass species for oribi in the high stocking rate camp. As a result, oribi adjusted their diet to the greatest degree in this camp incorporating previously avoided *Heteropogon contortus*, into their diet. Indeed, this grass species became one of the top contributing species in the diet of oribi that fed in this camp during the dry season (Table 1).

### Greenness and height of consumed grass species

During the wet season, both oribi and cattle only consumed very green (91–100%) grass, irrespective of stocking rates. However, the wet season feeding intensity in the high stocking rate camp reduced relative regrowth and influenced the availability of green grass for oribi in the dry season (Fig 2). Specifically, in the low and intermediate stocking rate camps, oribi had similar proportions of mainly green (0.65 and 0.64 respectively) and very green grass (0.35 and 0.36 respectively) in their dry season diet. Although oribi in the high stocking rate camp also consumed mainly green and very green grass, they focused primarily on mainly green grass (0.99), while the limited availability of very green grass prevented extensive use (0.01).

**Fig 3:**
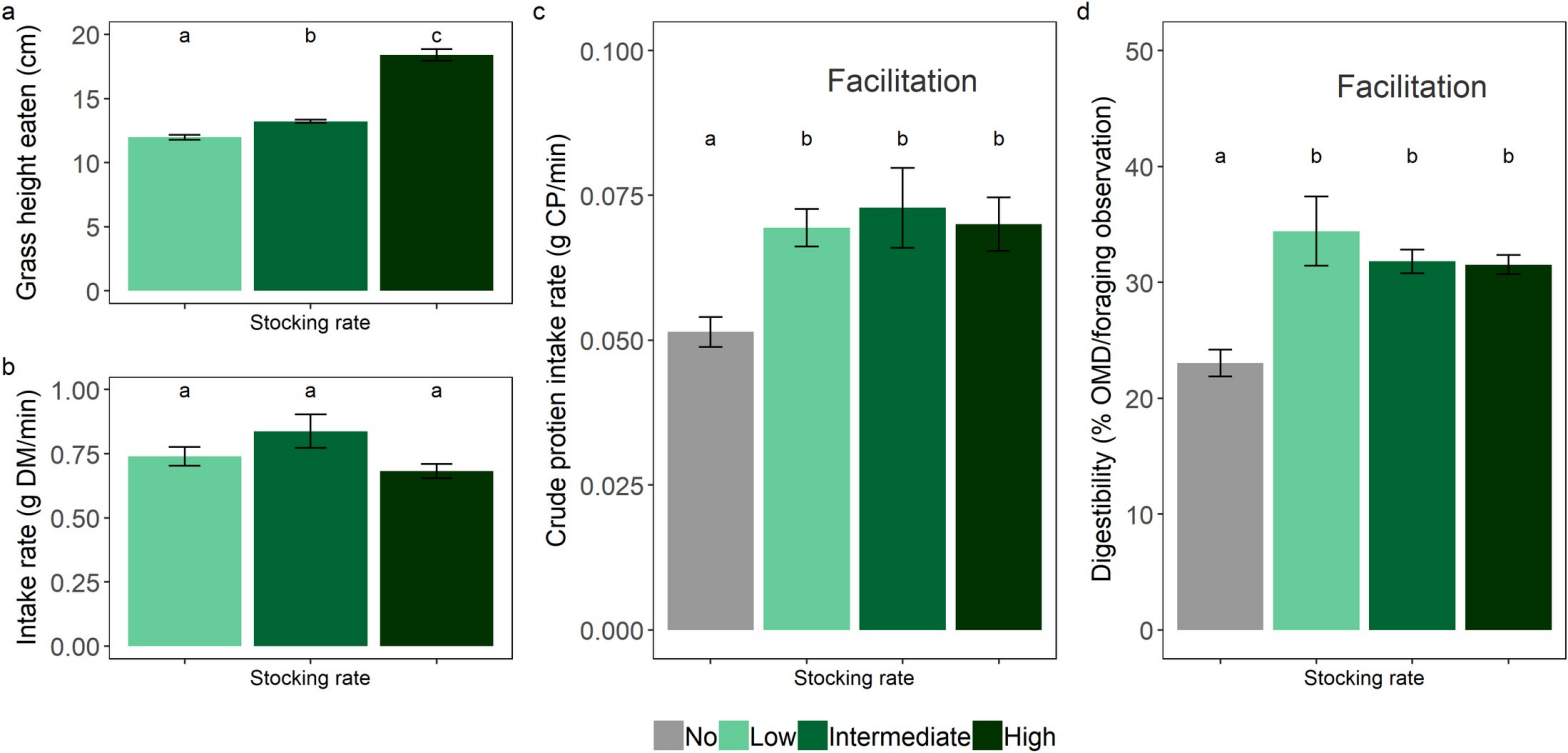
Wet season oribi antelope foraging metrics. Average (mean ± SE) for a) sward height eaten, b) intake rate, c) crude protein intake rate, and d) digestibility of consumed vegetation per foraging observation of oribi at low (1.7 ha per AU), intermediate (1.5 ha per AU) and high cattle stocking rates (0.95 ha per AU) during the wet season. The ‘No’ stocking rate in panels c and d are the crude protein intake rates that oribi obtained feeding in a camp without cattle. The difference in crude protein intake rates and the digestibility of consumed vegetation by oribi between when cattle are present or absent is the degree to which cattle facilitate oribi feeding. Letters denote significant differences.

Throughout the wet season, cattle fed on swards of similar height (mean ± SE) irrespective of stocking rates (11.83 ± 0.20 cm; GLM: *χ*^2^ = 0.102, *df* = 2, *P* = 0.950). However, the stocking rate of cattle influenced the sward height on which oribi fed (GLM: *χ*^2^ = 435.354, *df* = 2, *P* < 0.001; Fig 3A). Specifically, as the cattle stocking rate increased, oribi fed on taller grass swards (low: 12 ± 0.15 cm, intermediate: 13 ± 0.17 cm, high: 18 ± 0.31 cm).

**Fig 4:**
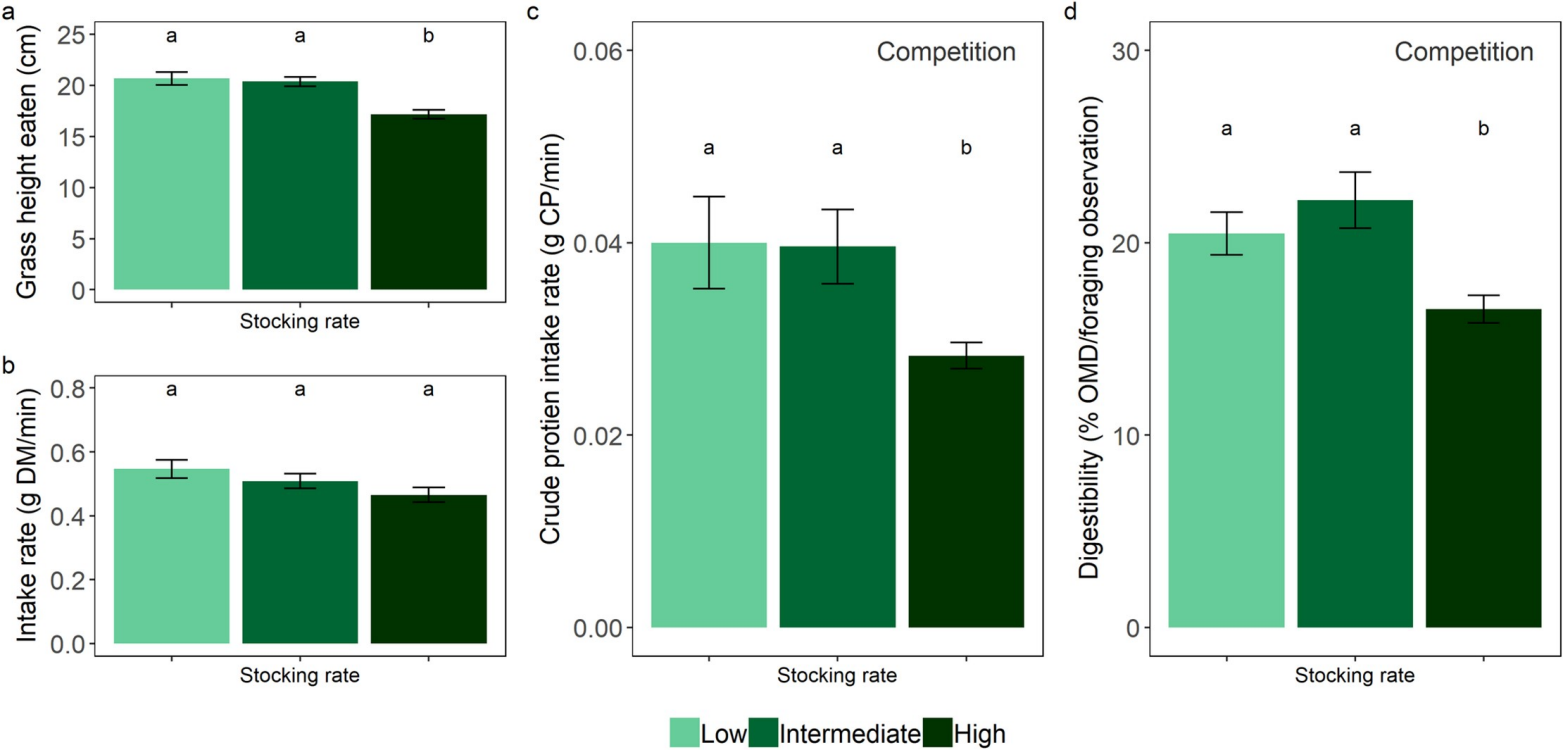
Dry season oribi antelope foraging metrics. Average (mean ± SE) for a) sward height eaten, b) intake rate, c) crude protein intake rate, and d) digestibility of consumed vegetation per foraging observation of oribi at low (1.7 ha per AU), intermediate (1.5 ha per AU) and high cattle stocking rates (0.95 ha per AU) during the dry season. The difference in crude protein intake rates and the digestibility of consumed vegetation by oribi between high cattle stocking rates and the lower cattle stocking rates is the degree to which cattle compete with oribi. Letters denote significant differences.

Although oribi and cattle did not directly interact during the dry season, the wet season foraging of cattle influenced the height of the grass swards on which oribi could feed (GLM: *χ*^2^ = 33.726, *df* = 2, *P* < 0.001; Fig 4A). In the low and intermediate stocking rate camps, oribi shifted and fed from taller grass swards (low: 20 ± 0.57 cm, intermediate: 20 ± 0.45 cm), compared to the wet season, while oribi in the high stocking rate camp continued feeding on tall grass swards (17 ± 0.43 cm).

### Nutritional intake rates of oribi

Despite feeding on different sward heights during the wet season, oribi maintained similar dry matter intake rates (GLM: *χ*^2^ = 4.750, *df* = 2, *P* = 0.093; Fig 3B), crude protein intake rates (GLM: *χ*^2^ = 0.228, *df* = 2, *P* = 0.892; Fig 3C), and consumed vegetation of similar digestibility (GLM: *χ*^2^ = 3.140, *df* = 2, *P* = 0.208; Fig 3D), irrespective of cattle stocking rate. However, oribi feeding with cattle were able to maintain higher crude protein intake rates (GLM: *χ*^2^ = 14.26, *df* = 1, *P* < 0.001; Fig 3C) and consume vegetation of higher digestibility (GLM: *χ*^2^ = 9.47, *df* = 1, *P* = 0.002; Fig 3D) during the wet season compared to oribi feeding without cattle.

During the dry season, when the cattle were not feeding in the camps, oribi fed on taller grass swards in all three camps and maintained similar dry matter intake rates (GLM: *χ*^2^ = 5.113, *df* = 2, *P* = 0.078; Fig 4B). However, the wet season cattle stocking rates did influence dry season crude protein intake rates (GLM: *χ*^2^ = 11.301, *df* = 2, *P* = 0.040; Fig 4C) and the digestibility of vegetation consumed by oribi (GLM: *χ*^2^ = 16.741, *df* = 2, *P* < 0.001; Fig 4D). A pairwise comparison of the marginal means revealed that oribi in the low and intermediate stocking rate camps obtained similar crude protein intake rates (*P* = 0.966) and consumed vegetation of similar digestibility (*P* = 0.295). Whereas, oribi in the high stocking rate camp obtained lower mean CP intake rates, and consumed vegetation that was less digestible compared to oribi in low (CP: *P* = 0.003; OMD: *P* = 0.002) and intermediate stocking rate camps (CP: *P* = 0.013; OMD: *P* < 0.001).

## Discussion

Conclusive evidence of competitive interactions between herbivores of different body size is scarce, particularly in an African context [1]. Here, we provide an experimental study that assessed how cattle foraging under different stocking rates influenced the foraging behavior of smaller-bodied oribi antelope and their ability to coexist with cattle. We found that (1) there is a high degree of dietary overlap between cattle and oribi; (2) this shared resource resulted in cattle facilitating oribi antelope when resources were abundant (wet season); (3) under high stocking rates, cattle reduced the availability of high quality resources; and (4) the combined resource overlap under high cattle stocking rates resulted in cattle competing with oribi when resources were limited (dry season). These competitive interactions occurred despite significant differences in body size and mouth morphology.

Cattle foraging under different stocking rates influenced grass greenness, grass regrowth, and ultimately the structural heterogeneity of available vegetation, with these variables having a negative relationship with increasing cattle stocking rates. Thus, cattle influenced oribi through bottom-up processes (i.e. availability of high-quality grass species–a limiting resource for oribi). In our study, cattle were managed under a continuous grazing policy, which allows for greater habitat heterogeneity compared to other more intensive practices such as rotational grazing of cattle [47]. This habitat heterogeneity allowed oribi to switch their foraging behavior (i.e. adaptive foraging options; [48]) depending on the conditions available to them. This suggests that, at least for oribi, structural heterogeneity in sward height, which is important for the maintenance of multiple herbivore guilds [20], may be an important resource to buffer the potential effects of competition and promote the coexistence of wildlife on livestock dominated rangelands [47, 49, 50].

During the wet season, in the high cattle stocking rate camp, we observed high dietary overlap between cattle and oribi as well as low levels of grass regrowth. These conditions suggest potential competition. Despite the high cattle stocking rate, this camp did maintain some degree of structural heterogeneity (47% CV). Thus, oribi feeding in the high cattle stocking rate camp (low grass regrowth) took advantage of the available structural heterogeneity and fed on taller swards compared to oribi under lower cattle stocking rates. The selective foraging of oribi allowed them to take advantage of the less intensively grazed areas by cattle and achieve similar crude protein intake rates and feed on vegetation of similar digestibility, despite feeding on taller vegetation, compared to oribi feeding under lower cattle stocking rates. Under low and intermediate cattle stocking rates, we observed high dietary overlap as well as an overlap between the height of consumed vegetation by oribi and cattle. Despite this, we found no evidence of competition at any cattle stocking rate during the wet season because oribi fed on high-quality grass regrowth that was generated by cattle grazing. In contrast, we found that oribi that fed with cattle achieved higher crude protein intake rates and consumed vegetation of higher digestibility compared to oribi that fed without cattle (Fig 3C and 3D). This highlights the important role that cattle can play in the conservation management of small-bodied wildlife on rangelands, where wild, large bulk-feeders, have been lost [26, 27]. Furthermore, the assumption that a high dietary overlap leads to competition has little validity and is likely a major contributor to the debate over the contentious issue of whether livestock compete with wildlife. Dietary overlap needs to be linked with nutritional intake rates, at the very least, to show potential competition, a step that is frequently overlooked in competition studies between wildlife and livestock [1].

During the dry season, oribi in the low and intermediate cattle stocking rate areas maintained a similar diet, but fed from taller vegetation (a previously unused resource), compared to their diet in the previous wet season. In contrast, oribi in the area with a high cattle stocking rate continued to feed on taller swards, but showed the highest degree of dietary expansion compared to their wet season diet. A high degree of dietary expansion during the dry season is consistent with predictions of a shared preference model that oribi are negatively affected by cattle grazing [49]. Under high stocking rates, cattle grazing in the previous wet season reduced the availability of high-quality grass species–a limiting resource for oribi. Thus, to compensate in the following dry season, oribi incorporated a previously avoided grass species, *H*. *contortus*, which has a lower nutritional quality compared to their preferred grass species, *H*. *hirta* and *T*. *triandra* (see S1 Table). Oribi also increased their use of the more palatable *H*. *hirta* and *T*. *triandra*. Despite this, oribi achieved lower crude protein intake rates and consumed vegetation of lower digestibility when compared to oribi feeding under lower cattle stocking rates. This suggests that for oribi, the cost of incorporating a low-quality grass species into their diet was greater than the benefits of somewhat increasing their use of more palatable grass species. These competitive effects occurred when cattle were not directly interacting with oribi (cattle were relocated to other grazing areas during the dry season). Thus, the previous wet season foraging by cattle caused cattle to compete with oribi through delayed habitat modification [1]. While the benefits of facilitation has been shown to be inter-seasonal (e.g. [51]), this is, to our knowledge, the first example of delayed, inter-season, competition by cattle on wildlife.

The net effects of interspecific interactions are the result of both competition and facilitation, with the net effect resulting from the interaction (competition or facilitation) that is quantitatively greater [8]. While the important role of competition in shaping communities has been well investigated, the perceived importance of facilitation in community-level processes has waned [52]. We propose that future research should address whether facilitation can offset the negative effects of competition and under what conditions this may actually occur. For example, African savannas or rangelands are characterized by a diverse grazing herbivore assemblage whose members vary in body size, dentition, and digestive physiology. These differences interact with forage quality to influence the degree to which competition or facilitation may structure the grazing community [53]. A number of studies posit that the beneficial effects of facilitation may offset the negative effects of competition [8, 23]. However, we argue that this is unlikely the case for small herbivores like oribi because the scaling of metabolic rates and the rates of body reserve depletion result in small-bodied species having a low resistance to starvation [54, 55]. Alternatively, oribi could increase the time they spent foraging. This response is likely to incur increased costs associated with heightened predation risk [56], which could be compounded by the low abundance of tall swards in high-stocking rate camps, which oribi use as refugia to avoid predation [38].

The cattle stocking rates for this study were not specifically selected, but rather occurred as a result of rangeland managers. Consequently, the similar foraging behavior of oribi, and the lack of any competition, in the low and intermediate stocking rates may be a result of the two cattle stocking rates being similar (1.7 ha per AU and 1.5 ha per AU). Alternatively, the selective foraging of oribi may buffer these small antelope from competition, such that there is a tipping point where competition occurs rather than a gradual response of oribi foraging behavior to increased cattle grazing (e.g. [6]). The cattle stocking densities for our study area fall within the recommended densities, however, across many African rangelands and communal grazing areas, cattle stocking densities greatly exceed recommended stocking densities [5]. Thus, it is plausible for competition to be more severe than presented in this study.

For oribi populations, and other wildlife in general, to be restored to sustainable levels, it is likely that these wildlife species will interact with cattle on shared rangelands (e.g. [16, 50, 57]). Private rangelands have been identified as important areas to support the long-term conservation of terrestrial fauna [58]. Our results, in conjunction with Odadi et al. [8] show that the coexistence of wildlife and cattle on rangelands has the potential to benefit both wildlife and cattle. However, to successfully integrate private rangelands into conservation and wildlife management, these systems need to be carefully managed [50]. For ecosystem stability, the strategic management of cattle densities is essential to firstly understand how the herbaceous layer responds to cattle grazing to ultimately reduce the competitive effects of cattle on wildlife, and secondly, to provide structural heterogeneity in sward height to sustain multiple herbivore guilds and promote biodiversity [59, 60]. Finally, we suggest that current management policies (e.g. avoiding temporal overlap in resource use during the dry season; [50, 61]) and differences in body size alone may not be entirely successful in mitigating competitive interactions between cattle and wildlife.

## Supporting information

S1 Table Seasonal mean % crude protein per grass species in different grass greenness categories across low (1.7 ha/AU), intermediate (1.5 ha/AU) and high (0.95 ha/AU) stocking rates at Arundel Farm, Ixopo, South Africa. (DOCX)
