# Supplementary material for: Coexistence between wildlife and livestock is contingent on cattle density and season but not differences in body size
Source: PLoS One. 2020 Jul 31;15(7):e0236895. doi: 10.1371/journal.pone.0236895 (PMC7394405; doi:10.1371/journal.pone.0236895)
Supplement: S1 Table — (DOCX) [file pone.0236895.s001.docx]

**Stears K, Shrader AM. Coexistence between wildlife and livestock is contingent on cattle density and season but not differences in body size. PLOS ONE**

S1 Table: Seasonal mean % crude protein per grass species in different grass greenness categories across low (1.7 ha/AU), intermediate (1.5 ha/AU) and high (0.95 ha/AU) stocking rates at Arundel Farm, Ixopo, South Africa.

| Season | Stocking rate | Species | Greenness (%) | N | Mean %CP | SE %CP |
| --- | --- | --- | --- | --- | --- | --- |
| Wet | Low | *Hyparrhenia hirta* | 91-99 | 5 | 8.1 | 0.18 |
|  |  | *Paspalum dilatatum* | 100 | 5 | 15.38 | 0.20 |
|  |  | *Setaria nigrirostris* | 100 | 5 | 12.06 | 0.52 |
|  |  | *Themeda triandra* | 100 | 5 | 8.09 | 0.15 |
|  |  |  |  |  |  |  |
|  | Intermediate | *Hyparrhenia hirta* | 91-99 | 5 | 8.22 | 0.11 |
|  |  | *Setaria nigrirostris* | 100 | 5 | 12.38 | 0.27 |
|  |  | *Themeda triandra* | 100 | 5 | 6.8 | 0.16 |
|  |  |  |  |  |  |  |
|  | High | *Hyparrhenia hirta* | 91-99 | 2 | 7.35 | 0.37 |
|  |  | *Setaria nigrirostris* | 100 | 5 | 12.37 | 0.48 |
|  |  | *Themeda triandra* | 100 | 5 | 8.84 | 0.10 |
|  |  |  |  |  |  |  |
|  | Ungrazed | *Hyparrhenia hirta* | 91-99 | 5 | 6.37 | 0.13 |
|  |  |  |  |  |  |  |
| Dry | Low | *Hyparrhenia hirta* | 76-90 | 5 | 5.65 | 0.14 |
|  |  |  | 91-99 | 5 | 7.6 | 0.23 |
|  |  | *Pennisetum clandestinum* | 91-99 | 3 | 14.1 | 1.86 |
|  |  | *Paspalum dilatatum* | 91-99 | 5 | 12.36 | 1.70 |
|  |  | *Themeda triandra* | 66-75 | 4 | 4.59 | 0.23 |
|  |  |  |  |  |  |  |
|  | Intermediate | *Hyparrhenia hirta* | 76-90 | 6 | 6.52 | 0.18 |
|  |  |  | 91-99 | 5 | 7.39 | 0.12 |
|  | |  |  |  |  |  |
| Table A1 continued | |  |  |  |  |  |
| Season | Stocking rate | Species | Greenness (%) | N | Mean %CP | SE %CP |
| Dry | Intermediate | *Pennisetum clandestinum* | 91-99 | 3 | 13.9 | 1.76 |
|  |  | *Paspalum dilatatum* | 91-99 | 5 | 12.22 | 1.70 |
|  |  | *Themeda triandra* | 66-75 | 4 | 4.49 | 0.21 |
|  |  |  |  |  |  |  |
|  | High | *Hyparrhenia hirta* | 66-75 | 4 | 5.67 | 0.25 |
|  |  |  | 76-90 | 5 | 7.74 | 0.14 |
|  |  | *Heteropogon contortus* | 76-90 | 3 | 3.46 | 0.45 |
|  |  | *Themeda triandra* | 66-75 | 3 | 5.71 | 0.59 |
